# Successful conservative treatment for massive tracheal necrosis after lung segmentectomy

**DOI:** 10.1186/s40792-023-01745-1

**Published:** 2023-09-11

**Authors:** Norifumi Tsubokawa, Takeshi Mimura, Kazuki Tadokoro, Yoshinori Yamashita

**Affiliations:** Department of General Thoracic Surgery, National Hospital Organization Kure Medical Center and Chugoku Cancer Center, 3-1 Aoyama-cho, Kure City, Hiroshima 737-0023 Japan

**Keywords:** Tracheal necrosis, Lung cancer, Esophageal cancer

## Abstract

**Background:**

Tracheal necrosis, which is rare because the trachea has rich in blood supply, can be a serious condition. Herein, we report the case of extensive tracheal necrosis that developed after right apical segmentectomy for a metastatic lung tumor of esophageal cancer.

**Case presentation:**

A 74-year-old man who had undergone thoracoscopic subtotal esophagectomy and gastric tube reconstruction via the posterior sternal route for esophageal adenocarcinoma 2 years previously was referred to our department with an enlarging nodal lesion in the right upper lung lobe. Computed tomography revealed a 30-mm tumor in the right apical segment with no lymph node enhancement, suggesting primary lung cancer or a metastatic lung tumor. The patient underwent right apical segmentectomy. The upper lobe was adherent to the chest wall and mediastinal fat from the apex of the lung to the dorsal side, with particularly strong adhesion at the esophagectomy site. After dissecting the adhesions, right apical segmentectomy was performed via complete video-assisted thoracic surgery. The patient was discharged on the 9th day after surgery without any complications. Pathologic findings revealed a metastatic lung tumor originating from the patient’s esophageal cancer. On the 26th day after surgery, the patient returned with dyspnea and increased sputum. Computed tomography images revealed that the posterior wall of the trachea was missing an area of 16 × 42 mm and was connected to the dead space after the right apical segmentectomy, with no effusion. We diagnosed extensive tracheal necrosis. Considering that the patient’s status was very well despite the extensive tracheal necrosis, we chose conservative treatment. After receiving 12 days of intravenous antibiotic treatment, his symptoms improved, and he was discharged on day 26 after admission.

**Conclusions:**

Right upper lung lobe resection after esophagectomy has a risk of tracheal necrosis. Conservative treatment is one approach to manage massive tracheal necrosis in patients with stable respiratory conditions.

**Supplementary Information:**

The online version contains supplementary material available at 10.1186/s40792-023-01745-1.

## Background

Tracheal necrosis is rare, with an incidence of 0.75% after esophagectomy because the trachea has rich blood supply [1, 2]. However, it can occur when this blood supply is disrupted and can be fatal in cases of respiratory failure or mediastinitis [3]. Tracheal necrosis developing after right lung resection for lung cancer is particularly rare. The treatment for tracheal necrosis can be decided based on the location and extent of the necrotic tracheal lesion [4]. Herein, we report the development of massive tracheal necrosis following right apical (S1) segmentectomy for a metastatic lung tumor of esophageal cancer. Conservative treatment using antibiotic therapy resulted in tracheal epithelialization.

## Case presentation

A 74-year-old man who underwent thoracoscopic subtotal esophagectomy and gastric tube reconstruction via the posterior sternal route 2 years previously for esophageal adenocarcinoma pT3N1M0 was referred to our department with an enlarging nodal lesion in the right upper lung lobe. Computed tomography (CT) revealed a 30-mm tumor in the right apical segment (S1) with no nodal enhancement, suggesting primary lung cancer or a metastatic lung tumor.

The patient underwent right S1 segmentectomy via complete video-assisted thoracic surgery (Additional file 1: Video S1). The upper lobe was adherent to the chest wall and mediastinal fat from the apex of the lung to the dorsal side, with particularly strong adhesion at the esophagectomy site. The adhesions were dissected, and the upper bronchus was identified from the dorsal side. However, we did not expose the trachea or its posterior wall. After dissecting the vessels and bronchus, the lung parenchyma was dissected using the energy device and staplers. The operation time was 220 min, and blood loss was 10 ml. Postoperative air leakage was not observed. The chest tube was removed on postoperative day (POD) 4, and the patient was discharged on POD 9 without any complications. Pathologic findings revealed a metastatic lung tumor originating from the patient’s esophageal cancer.

**Fig. 1:**
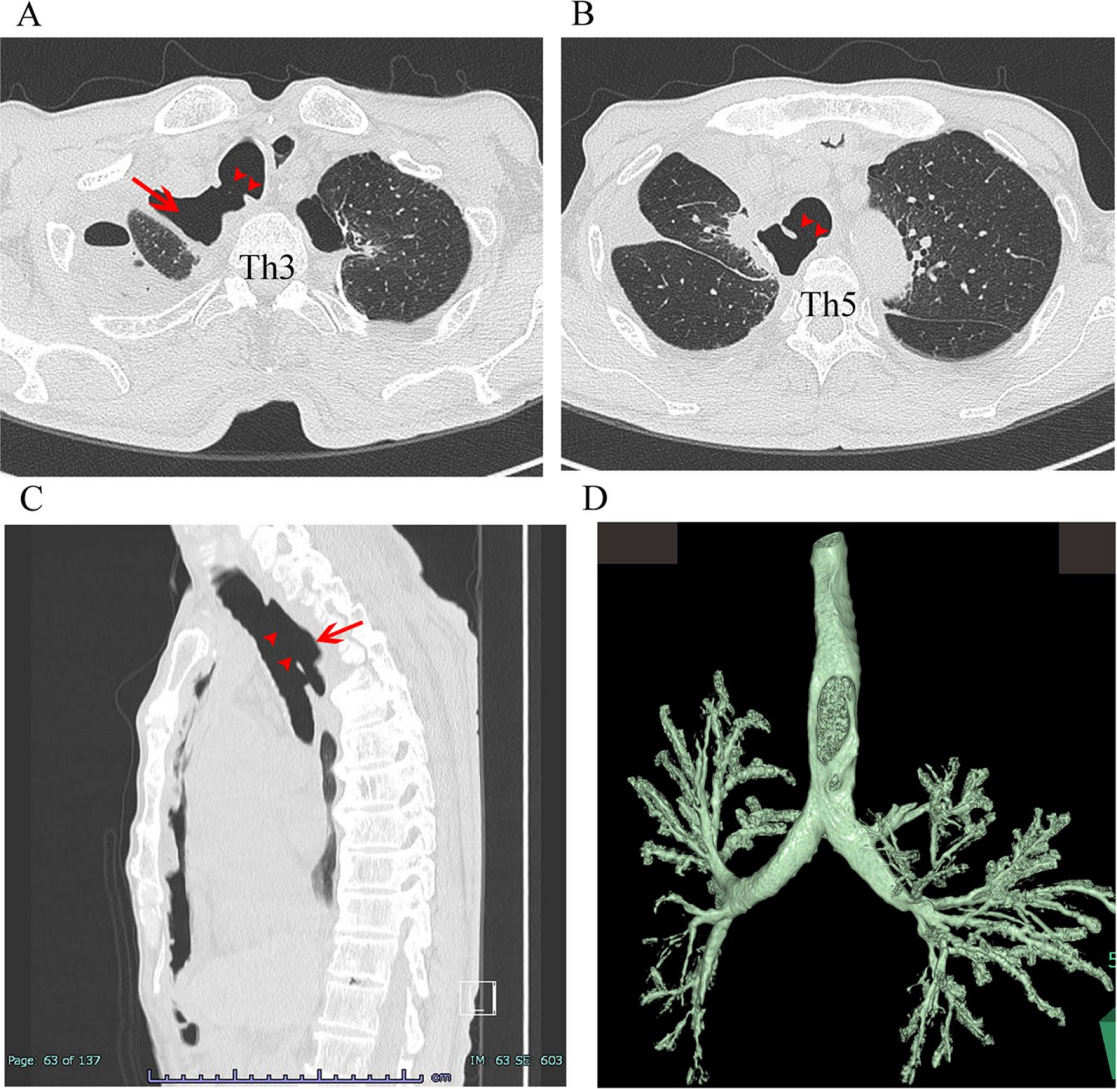
Initial chest CT findings of tracheal necrosis. Axial view at Th3 level (**A**) and Th5 level (**B**), sagittal view (**C**), and 3D-CT reconstruction image (**D**). The posterior wall of the trachea was missing an area of 16 × 42 mm (arrowheads) and connected to the dead space (arrow). *Th* thoracic vertebra, *CT* computed tomography

On POD 26, the patient returned with dyspnea and increased sputum. On admission, his body temperature was 37.3 °C and the percutaneous oxygen saturation was 91% on room air. His white blood cell count was 9300/μl, C-reactive protein 16.6 mg/dl, and serum albumin 2.9 g/dl. CT and 3D-CT reconstruction images revealed that the posterior wall of the trachea was missing an area of 16 × 42 mm and was connected to the dead space after the right S1 segmentectomy, with no effusion (Fig. 1, Additional file 1: Video S1). The bronchial stump was intact. We diagnosed extensive tracheal necrosis although bronchoscopy was not performed upon diagnosis because of its potential to worsen the tracheal necrosis. Considering that the patient’s status was incongruent with the extensive tracheal necrosis, we chose conservative treatment. We started oxygen therapy and administered intravenous antibiotics. In addition, through his enterostomy, enteral nutrition was started on the 3rd day after the onset of symptoms and was subsequently combined with oral intake for maintenance. After conservative treatment, there were improvements in dyspnea, sputum production, and inflammatory findings. His body temperature was 36.8 °C and the percutaneous oxygen saturation increased to 97% on room air. His white blood cell count and C-reactive protein decreased to was 4500/μl and 0.20 mg/dl, respectively, and serum albumin increased to 3.0 g/dl. He was discharged on day 26 after admission.

**Fig. 2:**
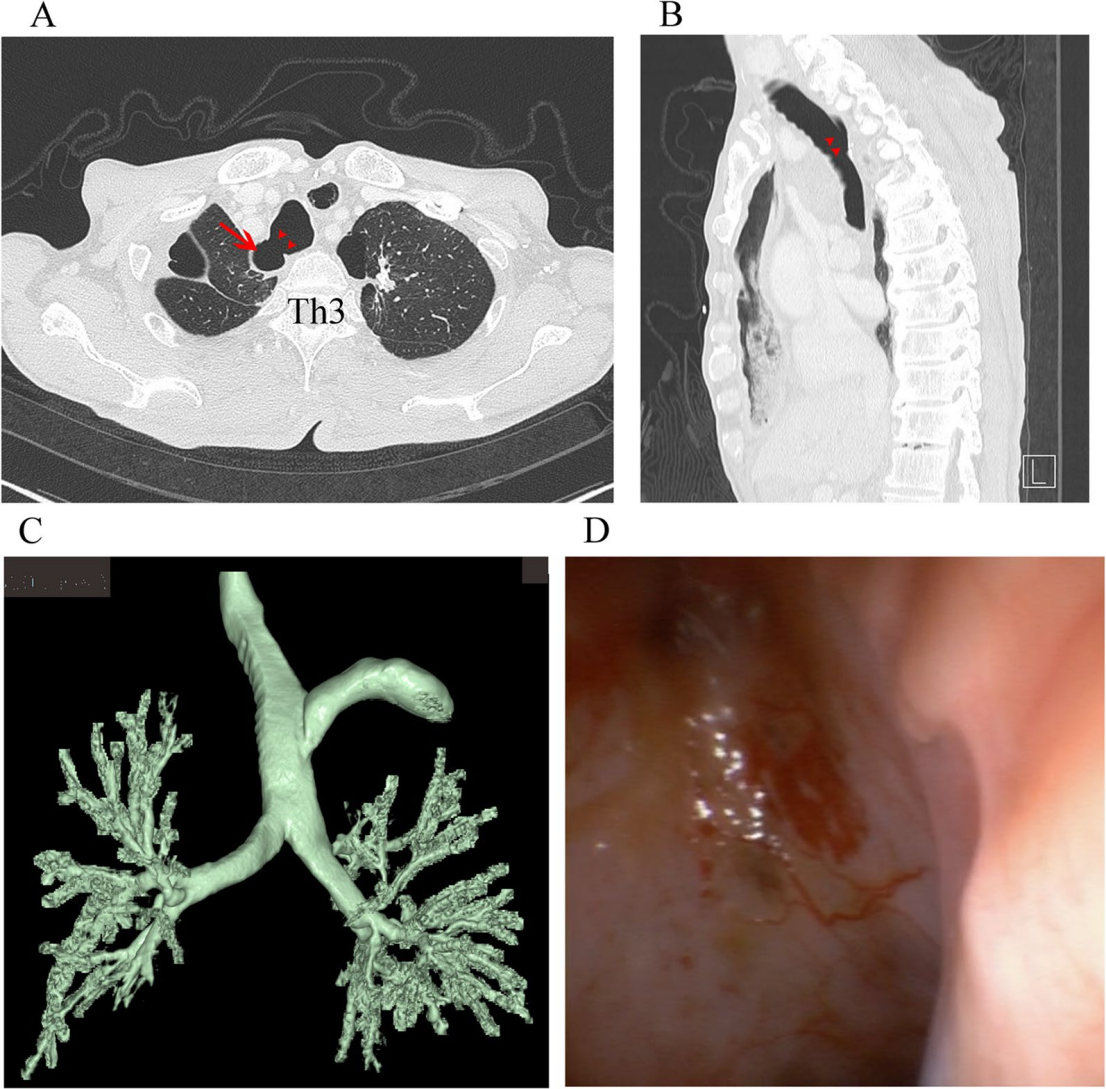
CT findings 6 months after receiving conservative treatment. Axial view at Th3 level (**A**), sagittal view (**B**), and 3D-CT reconstruction image (**C**). The area of defective posterior wall of the trachea (arrowheads) and the dead space (arrow) were significantly decreased. Bronchoscopy showed complete epithelialization of the trachea (**D**). *Th* thoracic vertebra, *CT* computed tomography

After 6 months, the patient underwent emergency small intestine resection for a strangulated ileus. CT showed a significant decrease in the defective area of the posterior wall of the trachea and the dead space in the right thoracic cavity (Fig. 2, Additional file 1: Video S1). Bronchoscopy showed complete tracheal epithelialization (Fig. 2).

## Discussion

The trachea is supplied by arterial blood that flows to the cervical and thoracic tracheal portions. These vessels are interconnected along the lateral surface of the trachea, providing a rich vascular network beneath the endotracheal mucosa [1, 4]. Tracheal necrosis occurs when this blood supply is disrupted (e.g., prolonged intubation, elevated cuff pressure, thyroid surgery, and tracheal injury or infection) [3]. In our case, the posterior wall of the trachea was completely exposed by esophagectomy, and then the capillaries entering the trachea were dissected during the right S1 segmentectomy, including dissection of adhesion around the trachea, which might have disrupted the tracheal blood supply.

Depending on the location and extent of the necrotic tracheal lesion, invasive surgery or stent replacement can be performed as treatment [3, 5, 6]. In our case, the CT scan showed a massive defect in the thoracic tracheal region, easily imaginable to be fatal, and we thoroughly discussed our treatment approach. There were no previous reports of such a case, and a direct closure would require a major musculocutaneous flap and placing the patient under long-term ventilatory control until the flap was fixed. However, because the patient’s respiratory state was surprisingly stable, we chose conservative treatment. In case the conservative treatment failed, we would have performed surgical repair with a latissimus dorsi muscle flap and placed the patient under long-term ventilator management and sedation to stabilize the repaired area. Fortunately, the conservative treatment worked. This could have been because the residual upper lobe preserved by the segmentectomy was large and almost adherent to the chest wall, thereby preventing lung collapse, and the remaining lung took the place of the posterior wall of the trachea, maintaining negative pressure in the thoracic cavity. Additionally, the drainage worked well because of the massive defects. Although the patient presented with an increased amount of sputum, there was no effusion in the dead space. Thus, the efficient drainage might have prevented mediastinitis. Although the patient’s nutritional status was poor owing to the esophagectomy and lung resection, he had received adequate nutritional support after tracheal necrosis through his enterostomy. Actually, his nutritional status improved; possibly, this nutritional support contributed to the successful conservative management of the tracheal necrosis.

## Conclusion

Thoracic surgeons should bear in mind that tracheal dissection after esophagectomy carries a risk of tracheal ischemia. Conservative treatment is one approach to manage massive tracheal necrosis in patients with stable respiratory conditions.

### Supplementary Information

**Additional file 1: Video S1**. Right S1 segmentectomy via complete video-assisted thoracic surgery. The patient had dyspnea and increased sputum 26 days after surgery. CT revealed that the posterior wall of the trachea was missing a massive section and connected to the dead space. The defective area and the dead space in the right thoracic cavity significantly decreased after conservative treatment. *CT* computed tomography.

## Data Availability

Not applicable.

## Abbreviations

CT: Computed tomography
POD: Postoperative day
